# Heavy Metal Loading Modifies Microplastic Mixture—Plant Interactions, Metal Availability, and Biochemical Responses in Aquatic Plants

**DOI:** 10.3390/toxics14090823

**Published:** 2026-09-16

**Authors:** Sandra Radić Brkanac, Marija Babić, Vibor Roje, Marko Rožman, Mario Janković, Emilie Kokić

**Affiliations:** 1 Department of Biology, Faculty of Science, University of Zagreb, Horvatovac 102A, HR-10 000 Zagreb, Croatia; mbabic@biol.pmf.hr (M.B.); jankovicmario49@gmail.com (M.J.); emiliekokic@hotmail.com (E.K.); 2 Department of Forestry, The Faculty of Forestry and Wood Technology, University of Zagreb, Svetošimunska 23, HR-10 000 Zagreb, Croatia; vroje@sumfak.hr; 3 Division of Physical Chemistry, Institute Ruđer Bošković, Bijenička 54, HR-10 000 Zagreb, Croatia; marko.rozman@irb.hr

**Keywords:** microplastics, metal release, adsorption, duckweeds, oxidative indicators

## Abstract

Microplastics (MP), including tire wear particles, are increasingly detected in aquatic environments, where they may interact with co-occurring contaminants such as heavy metals (HM). However, the influence of metal loading on MP–plant interactions and subsequent biological responses remains poorly understood. This study investigated how heavy metal loading modifies the behavior of mixed MP and their effects on two aquatic macrophytes, *Lemna minor* L. and *Spirodela polyrhiza* (L.) Schleid. Mixtures of polyethylene, polypropylene, polystyrene, and tire rubber particles were tested in both virgin and HM-loaded forms, with the latter preincubated with Cu, Zn and Pb. Duckweed species were exposed to MP concentrations ranging from 20 to 10,000 particles/L for seven days. HM-loaded MP released Cu and Pb into the plant-free exposure medium, whereas Cu release was also detected from the nominally virgin MP mixture. Cu accumulation occurred in both duckweed species, indicating transfer of MP-associated metals to aquatic plants. MP adsorption increased with exposure concentration and was higher in *L. minor* than in *S. polyrhiza*, with polypropylene showing the strongest association with plant surfaces. Metal loading reduced MP adsorption at intermediate concentrations, indicating that adsorbed metals can influence MP–plant interactions in a polymer- and concentration-dependent manner. Although MP exposure did not significantly affect growth, chlorophyll *a* content and antioxidant/detoxification enzyme activities were altered, particularly in *L. minor*. Overall, the results demonstrate that heavy metal loading modifies microplastic behavior and influences metal availability and sublethal responses in aquatic plants.

## 1. Introduction

Plastic pollution has resulted in the widespread occurrence of microplastics (MPs) in aquatic ecosystems [1]. Since MPs differ in polymer composition, size, morphology, surface properties, and degree of environmental transformation, they represent a heterogeneous group of particles with potentially different environmental behaviors and biological effects [1]. Many ecotoxicological studies have focused on individual polymer types and, in some cases, relatively high exposure concentrations [2,3]. However, environmental MP contamination generally comprises mixtures of different particle types. Consequently, investigating mixed particle mixtures is important for improving the environmental relevance of MP exposure assessments [4].

In addition to conventional polymers such as polyethylene (PE), polypropylene (PP), and polystyrene (PS), tire wear particles (R) are increasingly recognized as an important component of particulate pollution in aquatic environments [5]. Tire-derived particles differ from conventional plastic polymers because they consist of complex rubber matrices containing numerous additives and other constituents that may influence their environmental behavior and interactions with contaminants [5]. Environmental weathering and aging can modify MP surfaces and contribute to the release of chemicals associated with polymer matrices [6]. At the same time, fragmentation increases available surface area and number of reactive sites, potentially enhancing the adsorption of environmental contaminants, including heavy metals [1,7,8]. Thus, MPs can function not only as contaminants themselves but also as potential carriers or reservoirs for other pollutants such as heavy metals, with consequences for contaminant mobility, availability, and exposure pathways [9].

Aquatic plants and macrophytes are particularly relevant organisms for investigating these interactions because they are primary producers and can directly interact with suspended and floating particles [9]. Their relatively large surface area and continuous contact with surrounding water make them potential sites of MP adhesion and contaminant accumulation, and potentially important entry points for contaminant transfer through aquatic food webs. Duckweeds are therefore useful model organisms for investigating particle adhesion, contaminant accumulation, and early physiological responses to emerging pollutants [2,3]. Microplastic adhesion has been documented for several aquatic macrophytes, including *Lemna minor*, *Lemna minuta*, *Spirodela polyrhiza*, and *Cinclidotus aquaticus* [7,10,11,12]. Both *L. minor* and *S. polyrhiza* have also been shown to tolerate and accumulate metals to some extent [13], making these species suitable for investigating the combined occurrence of MPs and metal contaminants. MPs have been reported to affect plant growth, photosynthesis, and cellular metabolism, with growth-related parameters being the most frequently assessed endpoints [14].

Importantly, several studies have also investigated the combined effects of microplastics and heavy metals or other contaminants on aquatic plants. These studies demonstrate that co-exposure can modify metal accumulation and plant physiological or biochemical responses [12,15]. An additional limitation of the existing literature is that experimental studies have commonly focused on individual polymer types or microplastics added together with dissolved contaminants, whereas environmental microplastics occur as heterogeneous mixtures that may include conventional polymers together with tire-derived particles [4,5]. For example, in the studies of Yang et al. [16] and Farid et al. [17], microplastics were introduced into exposure systems containing dissolved heavy metals rather than being experimentally pre-loaded with metals before plant exposure. Moreover, relatively little attention has been given to whether metal loading changes microplastic retention on plant surfaces, metal release from particles, and subsequent metal accumulation in aquatic plants. Consequently, the extent to which the contaminant loading state alters the behavior of mixed microplastic particles and the exposure of aquatic plants to particle-associated metals remains insufficiently characterized.

To address this knowledge gap, the present study compares virgin and Zn-, Pb- and Cu-loaded mixtures composed of PE, PP, PS and tire rubber particles. These metals were selected based on their reported adsorption affinities for different MP polymers and waste tire rubber [18,19,20]. The study integrates measurements of metal release from the particles, microplastic adsorption/retention on *L. minor* and *S. polyrhiza*, metal accumulation in plant tissues, and plant physiological and biochemical responses. This approach allows us to distinguish responses associated with the presence of microplastic particles from those related to their heavy-metal loading and to determine whether contaminant loading modifies microplastic–plant interactions and metal transfer.

Accordingly, this study investigated whether heavy-metal loading modifies the behavior and biological effects of mixed MPs in duckweeds. Specifically, we aimed to: (1) characterize the release of Zn, Pb, and Cu from virgin and heavy-metal-loaded MP mixtures; (2) determine metal accumulation and potential transfer to duckweed tissues at environmentally relevant and higher, effect-oriented exposure concentrations [21]; (3) quantify MP adsorption to *L. minor* and *S. polyrhiza* and determine whether particle retention differs between virgin and heavy-metal-loaded mixtures, exposure concentrations, and particle types; and (4) assess species-specific physiological and biochemical responses, including growth, photosynthetic pigments, antioxidant enzyme activities, and oxidative damage markers. By integrating particle retention, metal mobility and accumulation, and biological responses within a single experimental framework, this study provides a more comprehensive assessment of how contaminant loading can modify MP–plant interactions and associated exposure pathways in aquatic systems.

## 2. Materials and Methods

### 2.1. Chemical Characterization of Microplastics and Rubber

Plastic pellets, namely polyethylene, polystyrene and polypropylene, were obtained from Europlast (Zagreb, Croatia). Original pellets (Φ 3–5 mm) were ground in liquid nitrogen and sieved to obtain the microplastic fraction up to 500 μm (90% of particles were in the range 100–500 μm). Particles were characterized by μRAMAN spectroscopy (HORIBA Jobin Yvon, Lille, France) (Figure S1). Recycled rubber granules (<2 mm) obtained from Gumiimpex—GRP d.o.o. (Varaždin, Croatia) were sieved to get the microrubber fraction up to 500 μm as previously described [12]. All particles were mixed in equal amounts and denoted as MP. The shape of individual polymer and rubber particles was evaluated using a light stereomicroscope Olympus BX-51 connected to a camera (Olympus DP70, Tokyo, Japan) at 100× magnification. Aside from PE particles, which were spherical, PP, PS, and R particles were irregularly shaped with sharp edges. The morphology of the particles is shown in Figure S2.

### 2.2. Plant Material and Experimental Design

For experiments, equal amounts of PE, PS, PP and R (1:1:1:1) microparticles were counted and weighed to achieve concentrations of 20, 100, 1000 and 10,000 particles/L, corresponding to 0.15, 0.74, 7.4 and 74 mg/L, respectively. To obtain heavy-metal-loaded (HM-loaded) MP, a portion of the particles was incubated in an aqueous solution containing 8 g/L of Cu, Pb, and Zn (CuSO_4_ × 5H_2_O, Kemika, Croatia; Pb(NO_3_)_2_, Alkaloid, Macedonia; ZnSO_4_ × 7H_2_O, Kemika, Croatia). MPs were incubated in a heavy metal mixture for seven days, as the adsorption equilibrium is typically reached within five days [9]. After incubation, HM-contaminated MPs were extracted from the solution by vacuum filtration (θ 47 mm, 0.5 μm Nucleopore membrane filters, Whatman^TM^, Marlborough, MA, USA), rinsed three times with distilled water to remove unadsorbed Cu, Pb, and Zn, and finally added to experimental nutrient solutions.

There were three experimental nutrient solutions: (1) P treatment—Steinberg nutrient medium with virgin MP in their final concentrations of 20, 100, 1000 and 10,000 particles/L (concentrations marked as P1, P2, P3 and P4, respectively); (2) M treatment—Steinberg nutrient medium [22] with mixture of HM-contaminated MP in their final concentrations of 20, 100, 1000 and 10,000 particles/L (concentrations marked as M1, M2, M3 and M4, respectively), and (3) control (C)—Steinberg nutrient medium without either virgin or HM-contaminated MP. Experiments were performed using two duckweed species—*L. minor* clone 9441 (Marburg, Germany) and *S. polyrhyza* (Botanical Garden, Zagreb, Croatia). Duckweeds from stock cultures (30 healthy plants of each species) were transferred to Erlenmeyer flasks containing Steinberg nutrient solutions. Duckweed toxicity tests were performed mostly following OECD [23] guidelines with slight modifications: the pH of a Steinberg nutrient solution was adjusted to 6.00, and plants were grown under fluorescent light with a 16 h photoperiod at 24 ± 2 °C. During the experiment, Erlenmeyer flasks were manually shaken (for 15 s twice a day for seven days).

### 2.3. Leaching Analyses

To evaluate the leaching of Cu, Pb, and Zn from HM-loaded MP into Steinberg nutrient medium, the highest concentrations of virgin (P4) and HM-loaded MP (M4) were incubated in the nutrient solution without plants for seven days. After seven days, aliquots of the nutrient solution were filtered into sterile Falcon tubes, acidified with 1% HNO_3_ (Carlo Erba Reagenti, Cornaredo, Italy), and stored at 4 °C before ICP-AES analysis (Thermo Fisher iCAP6300 Duo, Cambridge, UK). Measurements were performed in triplicate, and the results were expressed as μg/L.

### 2.4. Pb, Zn and Cu Analyses in Plant Material

Freeze-dried plant material was digested in Teflon cuvettes using HNO_3_ and 30% H_2_O_2_ (Carlo Erba Reagenti, Italy), heated at 200 °C in a microwave digestion system (Anton Paar Multiwave 3000, Anton Paar GmbH, Graz, Austria; rotor XFS-100), and then analyzed by ICP-AES. The concentrations of Pb, Zn, and Cu were determined in plants exposed to the highest concentration of virgin (P4) and to all concentrations of HM-loaded MP (M1-4), using a Multi-element Standard Solution (CPAChem, Bogomilovo, Bulgaria) and Multielement standard solution III for ICP (Fluka/Sigma-Aldrich, Buchs, Switzerland). Measurements were performed in triplicate, and the results were expressed as mg/kg on a dry weight (DW) basis.

### 2.5. Microparticle Detection and Small-Scale Adsorption Analyses by Optical Microscopy

To identify MP types and evaluate their adsorption onto the plant surfaces from the nutrient solution, ten randomly selected colonies (each consisting of two plants) were collected on day seven from each biological replicate (i.e., 30 colonies per treatment). The colonies were rinsed twice by immersing in dH_2_O and subsequently analyzed under a magnifying lens (40×). Adsorption capacity was expressed as the total number of adsorbed MPs per gram of fresh weight, as well as the number of adsorbed individual MP types (PP, PS, PE or R) on the same basis. During the analyses, both the type of adsorbed microparticle and the plant organ on which adsorption occurred were recorded.

### 2.6. Growth Rate Analyses

The growth of duckweeds was evaluated using frond number (FN) and fresh weight (FW) measured at the beginning (day 0) and at the end of the experiment (day 7). Based on the measured parameters, growth rates (GR) were calculated according to the following equation: GR = [ln x(t_2_) − ln x(t_1_)]/(t_2_ − t_1_), where x represents the observed parameter (FN or FW), t_2_ refers to the end of exposure (day 7), and t_1_ to the beginning of exposure (day 0) [23].

For subsequent toxicity testing, plants were freeze-dried (Alpha 1–2, Christ).

### 2.7. Photosynthetic Pigments Analyses

Chlorophyll and carotenoid concentrations were determined according to Wellburn [24]. Briefly, freeze-dried tissue samples were immersed in liquid nitrogen and finely ground (IST 400, Zagreb, Croatia). After adding 80% acetone, the samples were homogenized, centrifuged, and the absorbance of the supernatant was measured at 663, 646, and 470 nm using a Multiskan Microplate Photometer (Thermo Fisher Scientific, Waltham, MA, USA).

### 2.8. Oxidative Stress Analyses

Measured parameters were determined spectrophotometrically (Multiskan Microplate Photometer) using the previously reported methods [25]. For the antioxidant enzyme activity assessment, freeze-dried plant tissue was immersed in liquid nitrogen, finely ground (IST 400) and homogenized in 50 mmol/L K_2_HPO_4_/KH_2_PO_4_ buffer (pH 7.0) containing 0.1 mmol/L EDTA. The homogenates were centrifuged at 29,000× *g* for 30 min at 4 °C (Sigma 3K18, Osterode am Harz, Germany). Total soluble protein content was quantified according to Bradford [26] using bovine serum albumin as a standard. The assay is based on binding of Coomassie Brilliant Blue dye to proteins, with absorbance measured at 595 nm. Superoxide dismutase (SOD) activity was measured at 560 nm using nitroblue tetrazolium (Sigma-Aldrich). Catalase (CAT) activity was measured at 240 nm based on the decrease in H_2_O_2_ concentration. Ascorbate peroxidase (APX) activity was determined by monitoring ascorbate oxidation at 290 nm. Glutathione S-transferase (GST) activity was determined by measuring the glutathione-dependent increase in absorbance due to the conjugation of the CDNB substrate at 340 nm. The activities of all enzymes were expressed as units per milligram of protein (U/mg protein). Lipid peroxidation was determined by measuring malondialdehyde (MDA) content (nmol/g DW) at 532 nm using the thiobarbituric acid (TBA) assay. Protein carbonyl content was determined by measuring 2,4-dinitrophenylhydrazone formation at 370 nm and expressed as nmol/mg protein.

### 2.9. Statistical Analysis

All data are presented as mean values ± standard deviation. Two independent experiments were performed, each including three replicates per exposure concentration. Statistical analysis was performed using STATISTICA 14.0.1.25 (TIBCO, Inc., Palo Alto, CA, USA). Data normality was assessed using the Shapiro–Wilk test. When necessary, data were logarithmically transformed before statistical analysis. The effects of duckweed species, treatment, and their interaction on individual parameters were evaluated using two-way ANOVA. The statistical differences among the treatments were assessed by a one-way ANOVA followed by Tukey HSD test. In all the statistical analyses, the significance level was set at *p* < 0.05. Results marked with different letters indicate statistically significant differences among treatments.

## 3. Results

### 3.1. Metal Release from MPs to Nutrient Media and Metal Concentrations in Plants

The results show the release (leaching) of metals from HM-loaded (M treatment) and virgin MP (P treatment) into the nutrient medium before plant cultivation (plant-free media), indicating metal release from the MP themselves (Table 1). The highest concentrations of Cu and Pb were determined in the nutrient medium with the highest concentration of HM-loaded MP (M4), compared to the control and the nutrient medium containing virgin MP (P4). A significantly higher concentration of Cu compared to the control was also measured in the nutrient medium containing virgin MP. No significant differences in Zn concentration were observed among nutrient media with or without MP (C, P4 and M4).

**Figure 1:**
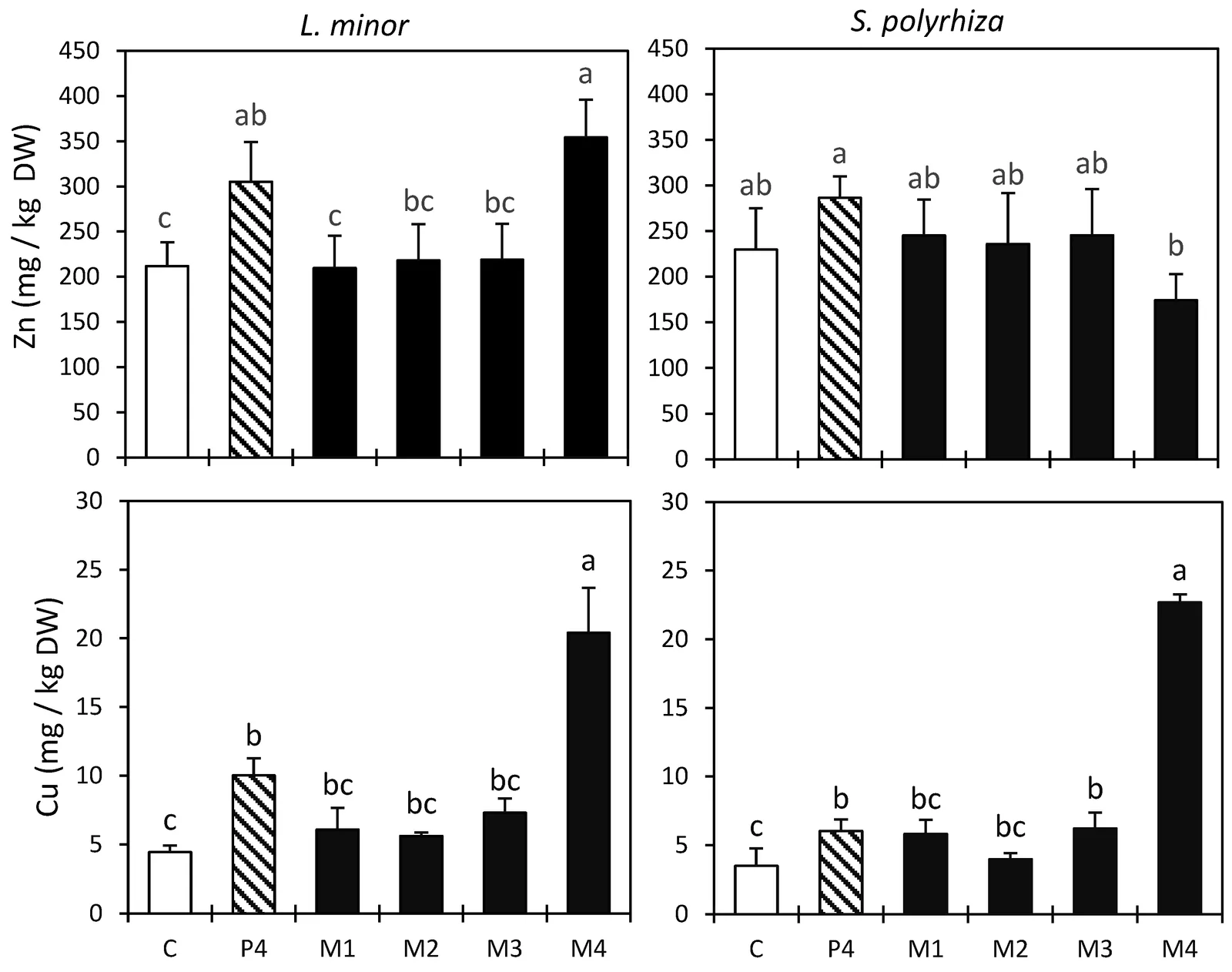
Contents of Zn and Cu in duckweeds treated with virgin (P treatments) or HM-loaded (M treatments) MPs mixture. Error bars denote standard deviations. Different letters indicate significantly different values among treatments at *p* < 0.05.

Nevertheless, Zn accumulated in significant amounts in *L. minor* under M4 treatment compared to the control and lower M treatment concentrations (Figure 1). Zn in *L. minor* also increased markedly under P4 treatment, showing a 47% increase compared to the control. Zn concentration in *S. polyrhiza* did not change significantly under any treatment compared to the control. However, the Zn concentration in *S. polyrhiza* exposed to the M4 treatment was significantly lower than that observed under the P4 treatment. Both duckweed species displayed a similar pattern of Cu accumulation, which increased under P4 treatment and was particularly elevated under M4 treatment compared to the control. The Cu concentration was two and four times higher under M4 treatment compared to P4 treatment for *L. minor* and *S. polyrhiza*, respectively (Figure 1). Regarding Pb, its concentration was below the detection limit (<1.41 mg/kg) in both duckweed species.

### 3.2. Microparticle Adsorption to Duckweeds

**Figure 2:**
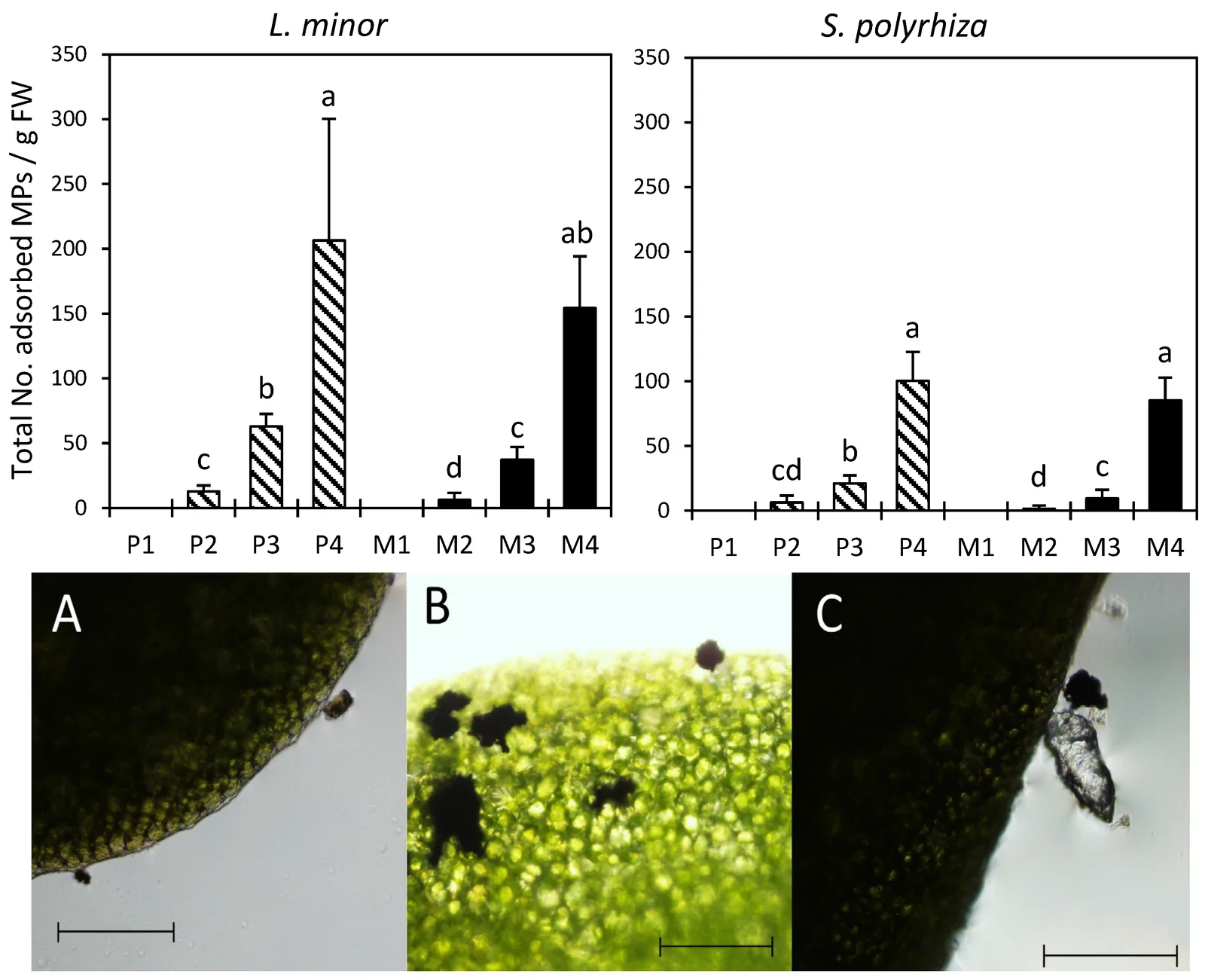
**Upper panel**: Total number of adsorbed microparticles to duckweeds exposed to virgin (P) or HM-loaded (M) MPs mixture. Error bars denote standard deviations. Different letters indicate statistically significant differences among significantly different values treatments at *p* < 0.05. **Lower panel**: (**A**) PP particles adhered to *L. minor*, (**B**) R particles adhered to *L. minor*, (**C**) PS and R particles adhered to *S. polyrhiza*. Bar = 200 μm.

The total number of adsorbed MPs (sum of all MP types), expressed per g FW (adsorption capacity), increased with increasing MP concentration, regardless of the treatment or duckweed species (Figure 2). No MP adsorption by duckweeds was detected in the control and at the lowest MP concentration.

**Figure 3:**
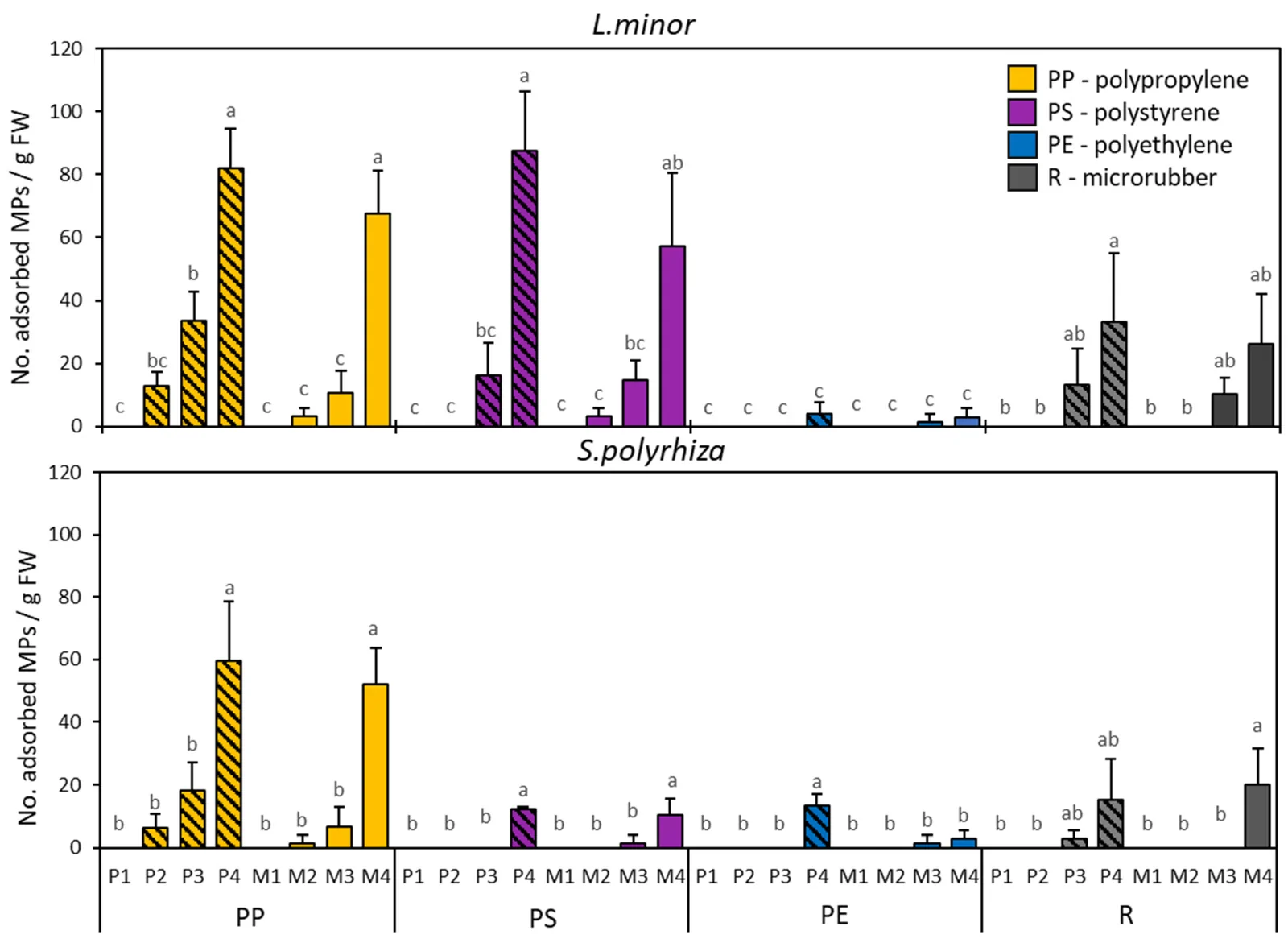
Number of adsorbed MP particle types in duckweeds exposed to different concentrations of virgin (P treatments) or HM-loaded (M treatments) MP mixtures. Error bars denote standard deviations. Different letters indicate significant differences among treatments at *p* < 0.05.

*L. minor* exhibited a markedly higher MP adsorption capacity than *S. polyrhiza* after seven days. The adsorption of virgin MP by *L. minor* was several times higher than that of *S. polyrhiza* at 100 and 1000 MP/L. Moreover, in the case of HM-contaminated MP (M treatment), the disparity in MP adsorption between the two duckweed species became even more pronounced at the respective concentrations. Unsurprisingly, the highest adsorption of virgin MP by duckweeds was observed at 10,000 MP/L (74 mg/L), reaching averages of 206 ± 93 and 100 ± 22 adsorbed MP in *L. minor* and *S. polyrhiza*, respectively. The average number of adsorbed HM-contaminated MP at the highest concentration was 154 ± 40 for *L. minor* and 85 ± 18 for *S. polyrhiza*. Total adsorption capacity of virgin MP to duckweeds, irrespective of the species, at 100 and 1000 MP/L (0.74 and 7.4 mg/L) was approximately two times higher than that of HM-contaminated MPs at the respective concentrations. However, the effect of treatment on MP adsorption by plants was less pronounced at 10,000 MP/L. With respect to MP type, *L. minor* (Figure 3) predominantly adsorbed PP and PS, particularly virgin MP, with a significant rise only at P3 treatment (100 MP/L). Similarly, *S. polyrhiza* showed the highest adsorption of PP compared to the other particle types present in the MP mixture, with slightly higher adsorption observed for virgin MP than for HM-loaded MP. The adsorption of virgin and HM-loaded PE and R particles by both duckweed species was similar. Regarding organ type, MP (particularly PP particles) were predominantly adsorbed onto the duckweed frond surface rather than the root (Figure S3).

### 3.3. Physiological and Oxidative Stress Parameters

For both parameters (FN or FW), the growth rate of *L. minor* in the control medium was approximately twofold higher than that of *S. polyrhiza* (Supplementary Table S1). Growth rates based on FN were 0.27 ± 0.012 and 0.15 ± 0.008 for *L. minor* and *S. polyrhiza* in control media, respectively. Regardless of concentration, exposure to virgin or HM-loaded MP mixture (P and M treatments) did not affect the growth rate of either duckweed species after seven-day exposure (Table S1).

**Figure 4:**
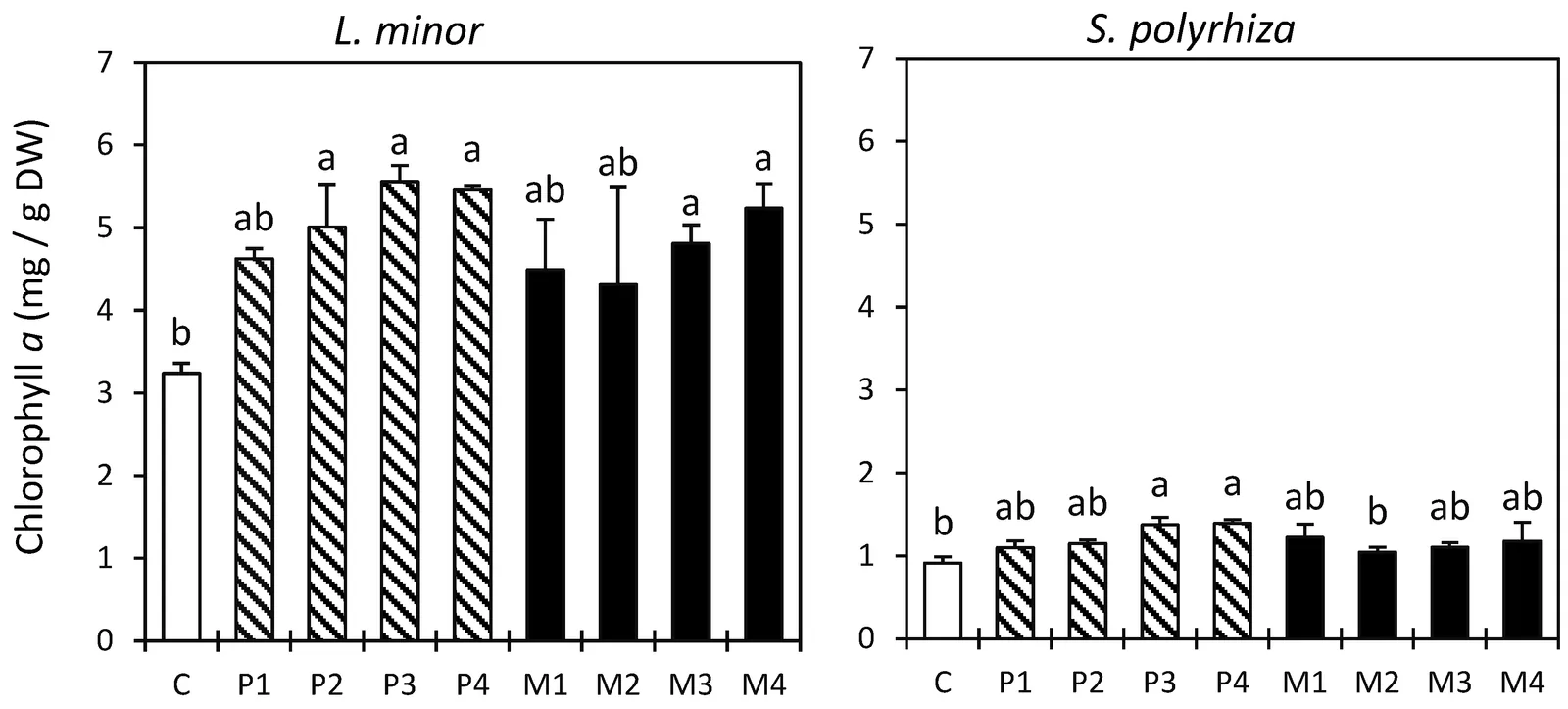
Chlorophyll *a* level in duckweeds treated with different concentrations of virgin (P) or HM-loaded (M) MP mixtures. Error bars denote standard deviations. Different letters indicate significant differences among treatments at *p* < 0.05.

Similar to growth, photosynthetic pigment levels were approximately threefold higher in *L. minor* than in *S. polyrhiza* cultivated in the control medium (Figure 4, Table S2). Chlorophyll *a* content in *L. minor* increased at P (42–70% compared to C) and M (33–62% compared to C) treatments (Figure 4), with significant effects observed at higher treatment concentrations. A similar trend was observed in *S. polyrhiza*, although the magnitude of chlorophyll *a* increase was lower (20–52% under P treatments and 15–34% under M treatments compared with C). Chlorophyll *b* and total carotenoid contents in *L. minor* were not influenced by P and M treatments, whereas in *S. polyrhiza* these pigments showed patterns similar to those of chlorophyll *a* (Table S2).

Unlike HM-loaded MPs, the virgin MP mixture significantly stimulated SOD activity in *L. minor* compared to the control (Table 2). Neither P nor M treatments affected SOD activity in *S. polyrhiza*. Both P and M treatments induced APX activity in *L. minor* compared to the control; however, the increase was not statistically significant at P1 treatment (Table 2). In *S. polyrhiza*, APX activity did not differ significantly from the control, although levels were significantly higher under the M3 and M4 treatments compared to the P1 and M1 treatments. CAT activity in *L. minor* was induced only by the M treatment, irrespective of concentration, compared to the control (Table 2). In *S. polyrhiza*, CAT activity under both P and M treatments was comparable to that of the control.

GST activity in *L. minor* started to decline from P2 and M1 treatments, with a significant decrease observed only at the M4 treatment (Table 2). In *S. polyrhiza*, GST activity decreased by 17–35% at P3 and P4 treatments and by 12–45% at M2–M4 treatments (Table 2). Indicators of lipid peroxidation (MDA) and protein oxidation (carbonyl content) were unaffected by either P or M treatments in both duckweed species (Table S3).

### 3.4. Effect of Duckweed Species and Treatments

Two-way analysis of variance (two-way ANOVA) was performed to evaluate the effects of duckweed species (*L. minor* and *S. polyrhiza*) and treatments with virgin or HM-contaminated MP mixture (P and M) on selected physiological and biochemical parameters, as well as on adsorption capacity (Table 3).

The analysis showed that all measured parameters were strongly affected by duckweed species (DS). Treatments (T) strongly influenced most of the measured parameters, except the growth rate based on FW, MDA and carbonyls. Almost all parameters were also found to be influenced by the interaction of duckweed species and treatments (DS × T), except growth rate based on FW, carotenoids, and carbonyl content. Notably, carotenoids were affected by both duckweed species and treatment, but showed no significant interaction between the two factors. Overall, the measured parameters showed different responses between the two duckweed species, regardless of treatment.

## 4. Discussion

### 4.1. Metal Release from MPs to Nutrient Media

The leaching analysis revealed significantly higher concentrations of Pb and Cu, compared to the control, in the nutrient media containing the highest concentration of HM-loaded MP (M4). These results suggest that the MP particles adsorbed metals, presumably in the form of metal complexes or cations [18], during a seven-day incubation in the HM solution and subsequently released them into the nutrient media. Among other factors, heavy metal concentration and MP properties (polarity, surface functional groups) appear to have the greatest influence on the MP adsorption capacities for heavy metals [27]. Godoy et al. [20] concluded that PP, PS, and especially PE used in their study had a negative charge and exhibited a high affinity for Pb and Cu. However, the extent of metal adsorption strongly depended on other MP properties, such as specific surface area, morphology, and porosity, as well as on pH value, the mineral composition of the aqueous solution and the presence of organic matter. Additionally, biofilm formation on MP surfaces was found to strongly influence metal adsorption by MPs [7].

We also observed a marked increase in Cu concentration in nutrient media containing virgin MP (P4). That result implies that the virgin MP contained Cu, either as an intentional additive or as a contaminant introduced during production. Metal-based compounds, including Cu, are commonly incorporated into plastic and rubber as stabilizers, catalysts, pigments, antimicrobial or cross-linking additives [6,28]. Regarding metal affinity, waste tire rubber showed effective adsorption of Zn and Pb, especially Cu, indicating its potential for wastewater treatment applications [19]. As metal release from individual MP types was not assessed in this study, it can only be hypothesized that rubber (R) particles within the MP mixture were the source of Cu leaching.

### 4.2. Metal Concentrations in Duckweeds

Cu accumulation in duckweeds under P4 and M4 treatments followed the pattern of Cu release from MPs in the corresponding plant-free treatments. Cu concentrations in plants were several-fold higher under M4 than under P4, indicating that Cu uptake by both duckweed species was driven by its availability in the nutrient medium. The strong accumulation of Cu released from either virgin or HM-contaminated MP observed in both duckweed species is consistent with previous findings. *L. minor* and *S. polyrhiza* exposed to considerably higher Cu concentrations (1.6–6.4 mg/L) have been reported to accumulate up to 1700 mg/kg DW of Cu [29]. Although Zn release from MP was not evident, *L. minor* accumulated considerable amounts of this micronutrient in both P4 and M4 treatments. Zn accumulation was over 20% higher in the M4 treatment than in the P4 treatment, although the difference was not statistically significant. Duckweeds are known to accumulate Zn efficiently, particularly under neutral pH conditions, temperatures above 21 °C and reduced nutrient availability [30]. Neither duckweed species accumulated detectable amounts of Pb, even at the M4 treatment. Although Pb is a nonessential element for plants, *L. minor* has been shown to efficiently accumulate Pb, Cu and other metals from multi-metal industrial effluents [13]. Because metal uptake was found to depend on metal availability and to decrease with increasing nutrient and organic matter content, the lack of Pb accumulation observed here may reflect the relatively low Pb concentration in the nutrient medium compared with Cu and Zn, even under the M4 treatment.

### 4.3. Adsorption of MPs to Duckweeds

Quantitative comparisons of plant MP adsorption are challenging due to the limited number of available studies [2], methodological differences in estimating adsorption capacity (e.g., per plant, surface area, fresh or dry weight), and variation in MP type, size and concentration among studies. In the present study, the highest adsorption of virgin MP was observed at 10,000 MP/L (74 mg/L), reaching approximately 200 MP/g FW in *L. minor* and 100 MP/g FW in *S. polyrhiza*. MP adsorption decreased markedly at environmentally relevant concentrations (20–1000 MP/L) and was undetectable at 20 MP/L. This is consistent with the results of Sfriso et al. [31], who reported that macrophyte species from the Adriatic Sea trapped up to 330 MP/g FW.

Comparing the effect of duckweed species, *L. minor* showed an overall higher adsorption capacity of MP than *S. polyrhiza*. The higher adsorption observed in *L. minor* may be explained by its higher surface-to-volume ratio and consequently greater surface area per unit biomass, resulting from its smaller frond size compared with *S. polyrhiza*. This is in agreement with a recently published study [32], which also indicates that morphological and chemical plant characteristics play an important role in providing available adsorption sites for MP. Both duckweed species have a glabrous, waxy cuticle with sporadic wax crystalloids on the adaxial frond surface, which creates a hydrophobic environment that likely contributes to MP adsorption [33,34]. The higher adsorption of MP to *L. minor* fronds may be partly explained by interspecific differences in epicuticular wax composition, which varies significantly among duckweed taxa [33]. Regarding MP type, both duckweed species showed the highest adsorption of PP particles, followed by R particles, while PE particles were the least adsorbed. The preferential adsorption of PP particles may be attributed to their irregular shape and high hydrophobicity [35]. PS particles, regardless of HM contamination, were more adsorbed by *L. minor* than by *S. polyrhiza*. This may be explained by the larger frond surface area and more textured micro-surface of *L. minor*, including pronounced venation ridges and papillae, which may enhance physical trapping of irregular, sharp-edged PS particles [33]. Although PE particles are also considered hydrophobic [35], their spherical shape reduces the effective hydrophobic contact area with the duckweed surface. Adsorption of R particles was lower than that of PP, likely due to negatively charged sulfonate and carboxylate groups derived from tire additives and vulcanization, which increase electrostatic repulsion from the negatively charged plant surface [5]. This is consistent with Rozman and Kalčikova [3], who reported weaker adhesion of tire particles to *L. minor* compared with irregular PE microbeads. However, both PE and R particles showed negligible adhesion at concentrations of 100 and 1000 particles/L. While limited evidence suggests that MP adsorption in aquatic plants is higher on roots than leaves [32], our results showed no organ-specific preference for PE and R particles, whereas PP and PS preferentially adhered to fronds. This likely reflects the affinity of hydrophobic, low-density PP and PS for the waxy frond surface, combined with buoyancy-driven contact with fronds. In the present study, preincubation of MP in a solution containing Cu^2+^, Zn^2+^, and Pb^2+^ significantly reduced their adsorption to both *L. minor* and *S. polyrhiza* at intermediate exposure concentrations, whereas no significant effect was observed at the highest concentration. Analysis of individual particle types suggested that this pattern was mainly driven by PP and PS particles in *L. minor* and PP particles in *S. polyrhiza*. However, differences between corresponding virgin and HM-loaded particles were not consistently significant for each particle type and exposure concentration. These findings suggest that the effect of HM loading on MP adsorption depends on exposure concentration, plant species and polymer type.

### 4.4. Physiological and Biochemical Responses in Duckweeds

The multiplication rate of *L. minor* in the control medium exceeded that of *S. polyrhiza*, suggesting that duckweed size may influence growth rate [36]. However, exposure to the MP mixture, regardless of concentration or treatment (P or M), did not affect the growth rate of either species after seven days. These findings are consistent with previous studies reporting no effects of various virgin or aged MP on duckweed growth and root development [2,10]. For example, *S. polyrhiza* exposed to spherical PP particles at concentrations up to 106 particles/mL for 5 days showed no inhibition of growth rate or root length [11]. In contrast, *L. minor* exposed to 100 mg/L PE fragments and tire wear particles for seven days exhibited reduced root length, while the specific growth rate remained unaffected [3]. Similarly, prolonged exposure of *L. minor* to irregular PE particles (100 mg/L) for 12 weeks did not affect the specific growth rate or root length during the final four weeks of exposure [37]. According to existing data, MP generally do not affect chlorophyll levels across different duckweed species [2,3,11,37].

In the present study, both duckweed species (especially *L. minor*) exhibited increased Cu uptake under P and M treatments, with significant accumulation observed at the highest exposure concentration. However, the accumulated Cu levels did not inhibit plant growth. Moreover, accumulated metals and/or plastic additives may reflect a stimulatory or adaptive response at the tested exposure conditions in the duckweeds, as reflected by elevated levels of photosynthetic pigments, especially chlorophyll *a*. A stimulatory effect of virgin MP was also observed on the chlorophyll *b* and carotenoid contents in *S. polyrhiza*. Although plastic additives (including metals) and small-sized MP particles at elevated concentrations can stimulate plant growth and enhance photosynthetic activity [38], similar effects were observed with low Cu concentrations, which may positively influence chlorophyll levels in duckweeds, as Cu functions as an essential micronutrient involved in electron-transfer processes during photosynthesis [39].

Changes in the activities of the assessed antioxidative and detoxifying enzymes reveal that MP and/or MP-derived leachates disrupted the redox balance in duckweed cells. Among the two plant species, *L. minor* exhibited a broader antioxidative response to the MP mixture than *S. polyrhiza*, as alterations in the activities of all measured antioxidative enzymes (SOD, APX and CAT) were detected in *L. minor*, whereas in *S. polyrhiza* only APX activity increased under M treatment. This difference may be attributed to the greater adsorption capacity of *L. minor*, resulting in more extensive contact with MP compared to *S. polyrhiza*. The induction of APX activity in response to both virgin and HM-preincubated MP mixtures in both species suggests a role in fine redox regulation, as APX, unlike CAT, is rapidly activated under low H_2_O_2_ concentrations [40]. Furthermore, the upregulation of SOD and CAT activities in *L. minor* exposed to P and M treatments, respectively, indicates H_2_O_2_ production occurring at different stages of exposure. SOD acts as the first line of defense by catalyzing the conversion of superoxide radicals into H_2_O_2_, whereas CAT efficiently scavenges excess H_2_O_2_ at higher concentrations. Consequently, HM-contaminated MP likely triggered an earlier increase in SOD activity, followed by a subsequent rise in CAT activity after seven days of exposure, whereas virgin MP and/or associated additives induced only SOD activity within the same exposure period. Moreover, GST, an enzyme involved in the detoxification of electrophilic compounds such as organic molecules and metals, exhibited a clear declining trend in response to both treatments in duckweeds, but especially under the M treatment. As GST utilizes glutathione as a reducing agent, the more pronounced alteration in its activity suggests a higher level of stress induced by HM-preincubated MP particles compared with virgin MPs [40].

Despite differences in antioxidative and detoxification responses between the two duckweed species, these mechanisms were sufficient to prevent oxidative damage, as no increase in lipid peroxidation or protein oxidation (assessed by MDA and carbonyl group content, respectively) was detected. This suggests that antioxidant defenses effectively maintained redox homeostasis under the applied treatments. Previous studies have reported no evidence of oxidative damage in *L. minuta* exposed to PS particles under chronic stress conditions [10], while combined exposure to PVC particles and Cd did not alter the antioxidative enzyme activities in *Vallisneria natans* (Lour.) after 14 days [41]. Consistent with our findings, low levels of MP and/or additive-induced stress have also been shown to stimulate the activity of antioxidant enzymes, including catalase or superoxide dismutase, in terrestrial plants [42].

Several limitations should be considered. The experiment was conducted under controlled laboratory conditions and focused on short-term exposure. Longer exposures, aging/weathering of MP and trophic transfer studies are required to determine whether the observed biochemical responses translate into population-level effects.

## 5. Conclusions

The enhanced leaching of Cu and Pb from HM-loaded MP indicates that MP may influence metal mobility and exposure pathways in freshwater ecosystems, although biological consequences depend on metal type, polymer characteristics and organism sensitivity. Both duckweed species accumulated Cu and adsorbed MP, with *L. minor* exhibiting greater adsorption efficiency than *S. polyrhiza*. These findings suggest that duckweeds can act as sinks for MP and associated contaminants, potentially influencing their retention and distribution in aquatic systems. However, MP adsorption to plant surfaces may represent a potential pathway for the transfer of MP and associated contaminants to higher trophic levels. HM loading reduced MP retention at intermediate exposure concentrations, with effects varying among polymer types. These findings demonstrate that contaminant loading can modify MP-plant interactions and emphasize the importance of evaluating environmentally relevant MP mixtures rather than pristine laboratory-generated particles alone. The higher MP retention observed in *L. minor* may have increased particle-associated exposure and contributed to the stronger biochemical responses detected in this species. Despite the absence of significant growth inhibition or oxidative damage during short-term exposure, alterations in antioxidative status indicate physiological responses to MP and/or their leached compounds. These findings suggest that mixed MP and associated contaminants can induce early biochemical adjustments before measurable adverse effects occur, highlighting the importance of longer-term studies to determine whether these responses translate into ecologically relevant consequences.

## Figures and Tables

**Table 1:** Concentrations of Cu, Pb and Zn in the plant-free control medium (C) and in plant-free media containing the highest concentration of virgin (P4) and HM-loaded MPs (M4).

	Cu (μg/L)	Pb (μg/L)	Zn (μg/L)
C	0.74 ± 0.062	0.93 ± 0.257	68.12 ± 6.192
P4	7.10 ± 0.429 *	1.54 ± 0.261	75.75 ± 6.000
M4	10.34 ± 0.670 **	3.61 ± 0.521 *	71.40 ± 2.432

Values represent the mean of three replicates ± SD. * and ** indicate significant differences within a column at *p* < 0.05 and *p* < 0.01, respectively, compared to the control.

**Table 2:** Superoxide dismutase (SOD; U/mg protein), ascorbate peroxidase (APX; U/mg protein), catalase (CAT; U/mg protein) and glutathione-S-transferase (GST; U/mg protein) activities in duckweeds exposed to different concentrations of virgin (P) or HM-loaded (M) MP mixtures.

	*L. minor*				*S. polyrhiza*			
Treatment	SOD	APX	CAT	GST	SOD	APX	CAT	GST
C	50.9 ± 6.98 b	7.10 ± 0.71 c	0.08 ± 0.006 b	0.57 ± 0.033 a	34.6 ± 2.99 a	28.7 ± 2.13 ab	0.03 ± 0.005 a	2.0 ± 0.18 a
P1	74.6 ± 3.43 a	8.67 ± 0.99 bc	0.11 ± 0.014 ab	0.59 ± 0.010 a	35.1 ± 8.61 a	27.1 ± 2.01 b	0.03 ± 0.013 a	2.2 ± 0.30 a
P2	65.8 ± 5.04 a	13.6 ± 0.80 a	0.11 ± 0.014 ab	0.52 ± 0.043 ab	32.3 ± 8.91 a	30.0 ± 1.36 ab	0.03 ± 0.002 a	1.9 ± 0.01 a
P3	68.7 ± 9.99 a	13.7 ± 2.82 a	0.09 ± 0.011 b	0.53 ± 0.023 ab	38.8 ± 0.41 a	29.7 ± 1.49 ab	0.04 ± 0.005 a	1.7 ± 0.34 ab
P4	71.3 ± 7.83 a	14.6 ± 0.69 a	0.09 ± 0.008 b	0.51 ± 0.075 ab	45.6 ± 1.90 a	29.4 ± 1.36 ab	0.03 ± 0.011 a	1.3 ± 0.21 bc
M1	47.0 ± 4.21 b	10.8 ± 1.94 ab	0.12 ± 0.016 a	0.54 ± 0.114 ab	32.9 ± 1.09 a	27.2 ± 5.77 b	0.03 ± 0.007 a	1.8 ± 0.27 ab
M2	48.0 ± 5.68 b	12.7 ± 1.01 a	0.13 ± 0.006 a	0.49 ± 0.007 ab	33.6 ± 7.16 a	29.7 ± 4.75 ab	0.03 ± 0.003 a	1.4 ± 0.27 bc
M3	53.2 ± 3.20 b	13.0 ± 0.89 a	0.13 ± 0.009 a	0.48 ± 0.056 ab	36.0 ± 1.70 a	36.9 ± 2.93 a	0.04 ± 0.002 a	1.3 ± 0.14 bc
M4	52.7 ± 4.45 b	15.7 ± 2.97 a	0.13 ± 0.005 a	0.38 ± 0.063 b	31.6 ± 5.91 a	36.6 ± 3.08 a	0.03 ± 0.004 a	1.1 ± 0.06 c

Values represent means of six replicates ± SD. Different letters within each column indicate significant differences among treatments at *p* < 0.05.

**Table 3:** Two-way ANOVA summary table showing the effects of duckweed species (DS), treatment (T), or interaction of duckweed species and treatment (DS × T) on adsorption capacity, physiological (growth rate, photosynthetic pigments) and biochemical (antioxidative enzymes, indicators of lipid peroxidation and protein oxidation) parameters in *L. minor* and *S. polyrhiza*.

	DS		T		DS × T	
	F	*p*	F	*p*	F	*p*
Growth rate—FN	1621.05	<1 × 10^6^	2.34	0.038	3.51	0.0043
Growth rate—FW	707.46	<0.000001	1.27	0.292	0.83	0.585
Chlorophyll *a*	1339.84	<0.000001	8.26	<0.00001	3.90	0.0021
Chlorophyll *b*	278.31	<0.000001	3.54	0.0041	2.70	0.0196
Carotenoids	861.24	<0.000001	2.37	0.037	1.13	0.367
SOD	224.49	<0.000001	8.41	<0.00001	3.99	0.0018
APX	722.86	<0.000001	6.23	<0.00001	2.24	0.0476
CAT	804.82	<0.000001	5.81	<0.00001	5.56	0.00013
GST	649.72	<0.000001	10.05	<0.00001	5.49	0.00015
MDA	162.31	<0.000001	1.49	0.196	2.68	0.0202
Carbonyls	800.85	<0.000001	0.80	0.606	0.47	0.870
Adsorption	17.09	0.0002	30.87	<0.000001	3.31	0.0062

Duckweed species *L. minor* and *S. polyrhiza* were exposed to P and M treatments (in four concentrations), and C treatment. The F values are given, and results were considered significant at *p* < 0.05.

## Data Availability

The original contributions presented in this study are included in the article/Supplementary Material. Further inquiries can be directed to the corresponding author.

## Supplementary Materials

The following supporting information can be downloaded at https://www.mdpi.com/article/10.3390/toxics14090823/s1, Figure S1: Representative Raman spectra of MPs: (A) polyethylene, (B) polypropylene and (C) polystyrene particles overlaid with their corresponding spectral-library matches; Figure S2: Morphology of the particles in the MPs mixture (PP—polypropylene, PS—polystyrene, PE—polyethylene, R—rubber) at 100x magnification. Bar = 200 μm; Figure S3: Relative distribution of adsorbed MPs between leaves and roots of duckweeds exposed to virgin (P) or HM-loaded (M) MPs mixture. Table S1: Growth rate based on FW and FN in *L. minor* and *S. polyrhiza* under control (C), virgin MPs mixture (P), and HM-loaded MPs (M) treatments. Table S2: Chlorophyll *b* and carotenoid contents in *L. minor* and *S. polyrhiza* under control (C), virgin MPs mixture (P), and HM-loaded MPs (M) treatments. Table S3: Malondialdehyde (MDA; nmol/g DW) and carbonyl groups (C=O; nmol/mg protein) in duckweeds exposed to different concentrations of virgin (P) or HM-loaded (M) MP mixtures.
