# Supplementary material for: Heavy Metal Loading Modifies Microplastic Mixture—Plant Interactions, Metal Availability, and Biochemical Responses in Aquatic Plants
Source: Toxics. 2026 Sep 16;14(9):823. doi: 10.3390/toxics14090823 (PMC13611071; doi:10.3390/toxics14090823)
Supplement: Supplementary file 1 [file toxics-14-00823-s001.zip › toxics-4549003-supplementary.pdf]

Supplementary materials

Heavy metal loading modifies microplastic mixture–plant interactions, metal availability, and biochemical responses in aquatic plants

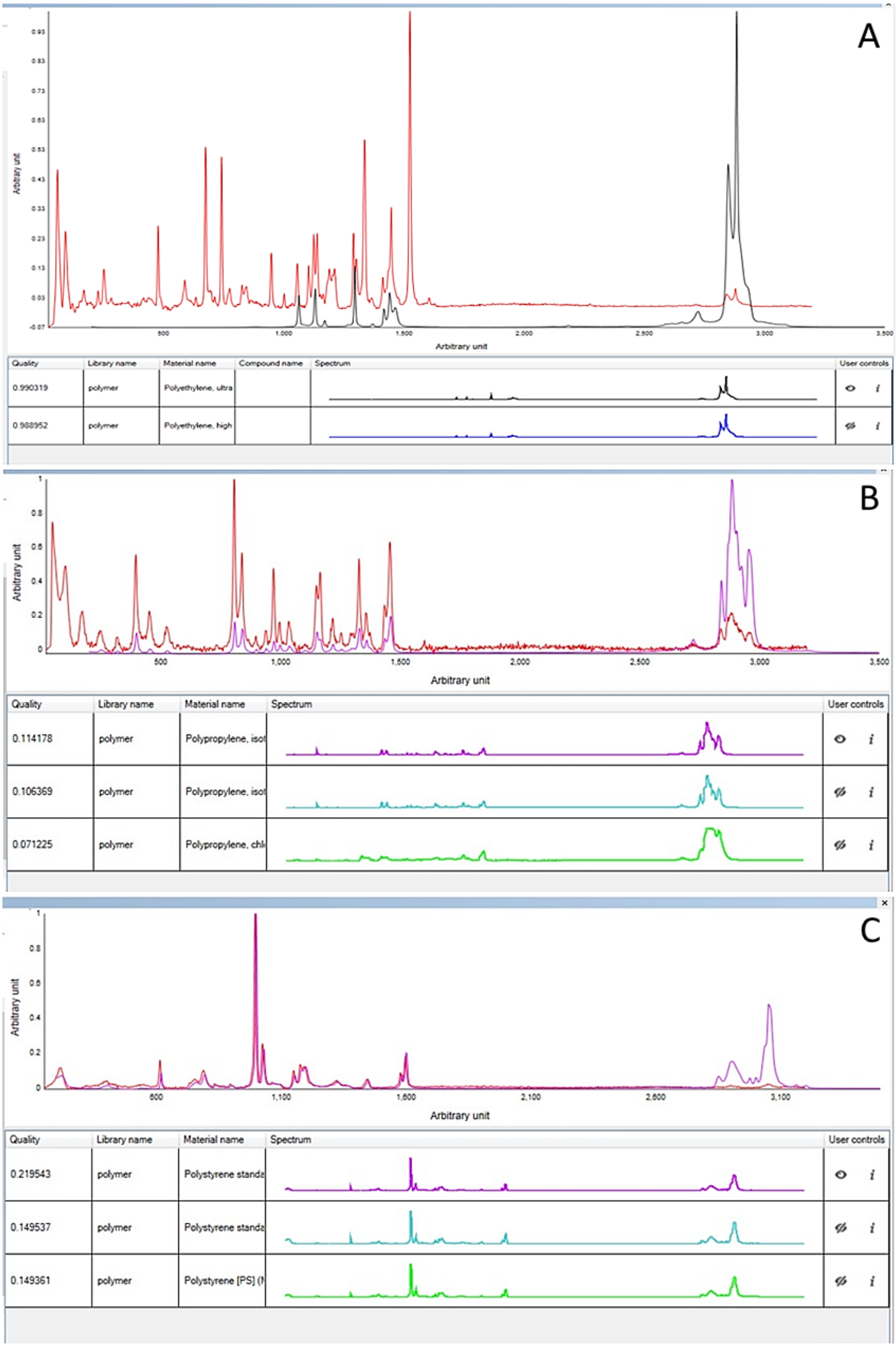

Figure S1. Representative Raman spectra of MPs: A) polyethylene, B) polypropylene and C) polystyrene particles overlaid with their corresponding spectral-library matches.

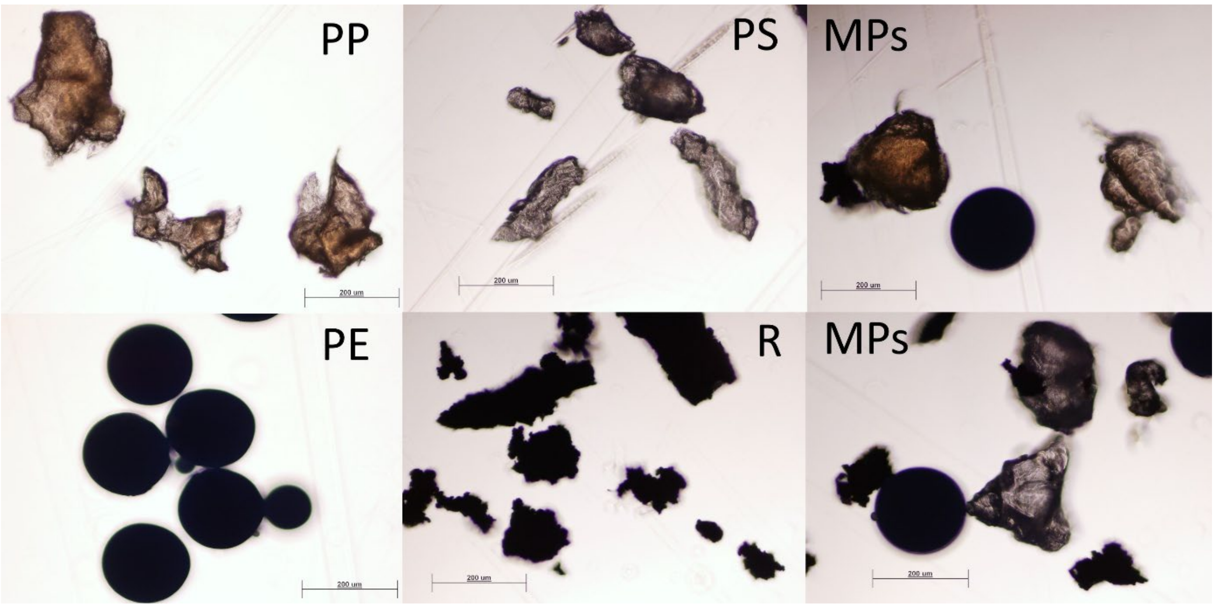

Figure S2. Morphology of the particles in the MPs mixture (PP - polypropylene, PS - polystyrene, PE – polyethylene, R – rubber) at 100x magnification. Bar = 200 μm.

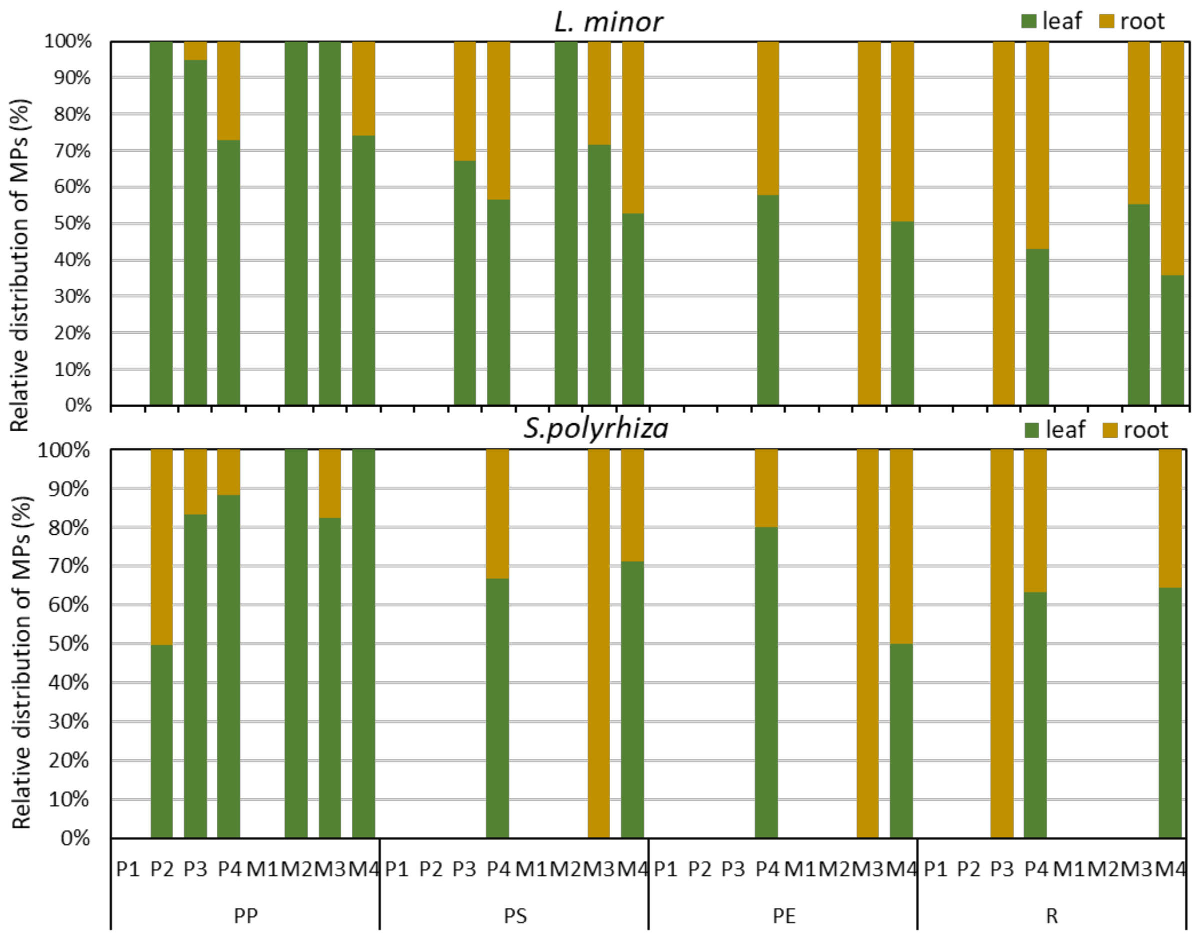

Figure S3. Relative distribution of adsorbed MPs between leaves and roots of duckweeds exposed to virgin (P) or HM-loaded (M) MPs mixture.

Table S1. Growth rate based on FW and FN in *L. minor* and *S. polyrhiza* under control (C), virgin MPs mixture (P), and HM-loaded MPs (M) treatments.

	L. minor		*S. polyrhiza*	
Treatment	Growth rate - FW	Growth rate - FN	Growth rate - FW	Growth rate - FN
C	0.29±0.015 a	0.27±0.012 a	0.13±0.013 a	0.15±0.008 a
P1	0.30±0.023 a	0.26±0.009 a	0.13±0.003 a	0.15±0.006 a
P2	0.31±0.024 a	0.26±0.008 a	0.12±0.005 a	0.15±0.007 a
P3	0.28±0.009 a	0.26±0.004 a	0.13±0.009 a	0.15±0.005 a
P4	0.27±0.012 a	0.25±0.003 a	0.12±0.019 a	0.14±0.003 a
M1	0.29±0.015 a	0.27±0.007 a	0.13±0.004 a	0.15±0.003 a
M2	0.33±0.077 a	0.29±0.026 a	0.13±0.004 a	0.14±0.008 a
M3	0.30±0.015 a	0.27±0.018 a	0.13±0.016 a	0.14±0.018 a
M4	0.31±0.010 a	0.29±0.011 a	0.13±0.005 a	0.15±0.009 a

Different letters within a column indicate significant differences at $p < 0.05$.
Data are expressed as mean ± SD, n=6.

Table S2. Chlorophyll *b* and carotenoid contents in *L. minor* and *S. polyrhiza* under control (C), virgin MPs mixture (P), and HM-loaded MPs (M) treatments.

	L. minor		*S. polyrhiza*	
Treatment	Chlorophyll *b* mg / g DW	Carotenoids mg / g DW	Chlorophyll *b* mg / g DW	Carotenoids mg / g DW
C	1.20±0.148 a	1.49±0.144 a	0.28±0.013 b	0.35±0.030 b
P1	0.92±0.571 a	1.54±0.077 a	0.35±0.026 ab	0.40±0.025 ab
P2	1.80±0.308 a	1.69±0.222 a	0.36±0.023 ab	0.42±0.027 ab
P3	1.90±0.141 a	1.78±0.039 a	0.44±0.029 a	0.50±0.040 a
P4	1.72±0.384 a	1.95±0.001 a	0.44±0.007 a	0.50±0.009 a
M1	1.33±0.185 a	1.47±0.379 a	0.38±0.049 ab	0.43±0.048 ab
M2	1.66±0.587 a	1.73±0.447 a	0.32±0.022 b	0.38±0.004 b
M3	1.68±0.044 a	1.64±0.050 a	0.35±0.022 ab	0.40±0.013 ab
M4	1.08±0.072 a	1.85±0.139 a	0.36±0.072 ab	0.42±0.077 ab

Different letters within column indicate significant differences at $p < 0.05$.
Data are expressed as mean ± SD, n=6.

Table S3. Malondialdehyde (MDA; nmol / g DW) and carbonyl groups (C=O; nmol/mg protein) in duckweeds exposed to different concentrations of virgin (P) or HM-loaded (M) MP mixtures.

	L. minor		*S. polyrhiza*	
Treatment	MDA	C=O	MDA	C=O
C	0.12±0.003 a	2.6±0.30 a	0.16±0.023 a	1.1±0.14 a
P1	0.12±0.012 a	2.6±0.34 a	0.18±0.005 a	1.2±0.07 a
P2	0.13±0.018 a	2.8±0.27 a	0.18±0.040 a	1.1±0.16 a
P3	0.12±0.018 a	2.6±0.14 a	0.19±0.025 a	1.1±0.09 a
P4	0.12±0.007 a	2.6±0.39 a	0.16±0.032 a	1.2±0.05 a
M1	0.11±0.005 a	2.4±0.23 a	0.22±0.017 a	1.1±0.09 a
M2	0.10±0.005 a	2.5±0.03 a	0.21±0.002 a	1.1±0.04 a
M3	0.12±0.033 a	2.6±0.08 a	0.21±0.031 a	1.1±0.02 a
M4	0.11±0.002 a	2.7±0.23 a	0.17±0.015 a	1.3±0.12 a

Different letters within a column indicate significant differences at $p < 0.05$. Data are expressed as mean ± SD, n=6.
